# Automated Aortic Quantification Based on Artificial Intelligence: Validation Using Contrast-Enhanced and Non-Contrast CT Scans from the Same Session

**DOI:** 10.3390/bioengineering13040471

**Published:** 2026-04-17

**Authors:** Jia-Sheng Hong, Yun-Hsuan Tzeng, Kuan-Ting Wu, Shih-Yu Huang, Ting-Wei Wang, Guan-Yu Li, Chun-Yi Lin, Ho-Ren Liu, Hai-Neng Fu, Yung-Tsai Lee, Wei-Hsian Yin, Yu-Te Wu

**Affiliations:** 1 Institute of Biophotonics, National Yang Ming Chiao Tung University, Taipei 112, Taiwan; eternity.jshong@nycu.edu.tw (J.-S.H.);; 2 Health Management Center, Cheng Hsin General Hospital, Taipei 112, Taiwan; 3 School of Medicine, College of Medicine, National Yang Ming Chiao Tung University, Taipei 112, Taiwan; 4 Heart Center, Cheng Hsin General Hospital, Taipei 112, Taiwan; 5 Department of Exercise and Healthy Science, National Taipei University of Nursing and Health Science, Taipei 112, Taiwan; 6 Brain Research Center, National Yang Ming Chiao Tung University, Taipei 112, Taiwan; 7 College Medical Device Innovation and Translation Center, National Yang Ming Chiao Tung University, Taipei 112, Taiwan; 8 Center for Smart Health and Medicine, Taipei City Hospital, Taipei 112, Taiwan

**Keywords:** automated aortic quantification, artificial intelligence, computed tomography, aortic dilation, contrast-enhanced CT, non-contrast CT, TotalSegmentator, 3D Slicer, open-source workflow

## Abstract

Early detection of aortic dilatation is clinically important for preventing progression to serious aortic disease and enabling timely intervention. We aimed to develop an AI method for quantifying the aorta in both contrast-enhanced and non-contrast CT scans, assisting early detection of aortic dilation. A total of 190 patient cases were analyzed, each having paired contrast-enhanced and non-contrast CT scans acquired in the same session, resulting in 380 scans. Our approach, based on open-source tools, demonstrated strong agreement with manual annotations, particularly in the ascending aorta. For contrast-enhanced CT, the AI achieved a correlation coefficient of 0.987 and intraclass correlation coefficient (ICC) of 0.986; for non-contrast CT, both were 0.945. Compared with clinical records, the sensitivity of AI detection was 97% for contrast-enhanced CT and 94% for non-contrast CT. This AI-based workflow enables highly sensitive automated aortic quantification in both contrast-enhanced and non-contrast CT scans, supporting broader clinical applicability across different imaging conditions.

## 1. Introduction

As the primary artery, the aorta is pivotal in transporting oxygen-rich blood from the heart to the rest of the body, ensuring body organs receive the necessary oxygen for survival [1]. Its structure is vital for the integrity of this major blood vessel, with issues such as aneurysms and dissections posing significant life-threatening risks [2,3]. Aortic dilation is a crucial precursor to aortic aneurysm or dissection, or in patients with aortic valve disease, requiring treatment intervention once surpassing certain thresholds [4,5]. The prevalence of aortic dilation ranges from 1.4% to 23%, varying across different study populations [6,7,8]. Aortic dilation is related to ageing and existing cardiovascular diseases or abnormalities, leading to an elevated risk of subsequent aortic disease [9].

Advanced diagnostic techniques, such as echocardiography, MRI, and CT, enable early detection of aortic anomalies, ensuring timely treatment [10,11]. Following the guidelines, early surgical or pharmacological interventions have significantly enhanced patient prognosis, reducing the incidence of catastrophic outcomes [4,5]. Early detection and monitoring of aortic diseases can improve patient outcomes and minimize societal costs [12]. Although contrast-enhanced CT remains important for vascular evaluation, the use of intravenous contrast media in routine CT practice still involves practical and safety considerations, and prior studies have examined the renal effects of different iodinated contrast agents in outpatients undergoing CT [13]. Non-contrast CT scans, such as those performed in lung cancer screening [14] as part of health screening programs, often include aortic imaging, offering an opportunity for early detection of aortic abnormalities. Maximizing the utility of any CT examination can significantly contribute to the early detection of diseases.

Artificial Intelligence (AI) techniques have rapidly advanced, enabling numerous automated analyses across medical imaging applications, including disease detection, classification, and quantitative assessment [15,16]. In parallel, recent studies have continued to propose new hybrid neural network architectures and attention-based frameworks for complex pattern recognition tasks [17], while growing attention has also been given to model interpretability and feature contribution analysis in AI-assisted clinical decision systems [18,19]. In clinical AI applications, the variance in imaging techniques can pose challenges [20]. With its quantitative Hounsfield Units (HU), CT imaging is suitable for clinical AI applications. TotalSegmentator [21], introduced in early 2022, is a powerful AI model capable of segmenting various organs in CT images, advancing AI applications in CT imaging, including automated aortic segmentation [22,23,24,25,26,27,28,29,30,31]. However, the original TotalSegmentator output provides only a single aorta label and does not by itself perform the subsequent post-processing steps required for aortic quantification, such as straightening, centerline-based analysis, and cross-sectional measurement. While TotalSegmentator can automatically segment various tissues and organs in CT, a detailed and validated process for aortic quantification remains lacking. Should a method for quantifying the aorta be developed based on it and undergo proper validation, it would greatly benefit clinical diagnosis and monitoring.

Previous studies have shown that standardized and semi-automated measurement strategies, including double-oblique short-axis and semiautomatic centerline analysis, can improve the repeatability and reproducibility of thoracic aortic diameter assessment on CT [32]. More recently, fully automated methods have been developed for aortic diameter measurement in both ECG-gated contrast-enhanced CT angiography and non-ECG-gated non-contrast CT, demonstrating the feasibility of automated thoracic aortic quantification in both dedicated vascular imaging and screening-oriented settings [31,33]. In parallel, broader quantitative imaging studies, including preclinical vascular imaging and radiomics research, have suggested that imaging-derived vascular features may serve as noninvasive biomarkers for characterizing arterial remodeling and supporting translational investigation [34,35]. However, despite these advances, clinically validated automated aortic quantification across both contrast-enhanced and non-contrast CT scans, particularly using paired scans acquired from the same patient during the same imaging session, remains limited.

The primary innovation of the present study lies not in proposing a fundamentally new deep neural network architecture, but in developing a practical AI-enabled medical imaging workflow for automated aortic quantification based on open-source models and tools. Specifically, we integrate whole-aorta segmentation with geometry-aware post-processing steps, including centerline extraction, vessel straightening, resampling, and cross-sectional diameter/area calculation, within an installable 3D Slicer module. This system-integration design is intended to support early detection, quantitative assessment, practical reproducibility, and validation across both contrast-enhanced and non-contrast CT settings.

Our research aims to develop and validate a fully automated aortic quantification workflow using TotalSegmentator as the core segmentation model within an open-source pipeline. The workflow was evaluated using paired contrast-enhanced and non-contrast CT scans acquired from the same patient during the same imaging session to assess its feasibility, robustness, and clinical applicability across contrast conditions.

## 2. Materials and Methods

### 2.1. Study Population

**Figure 1:**
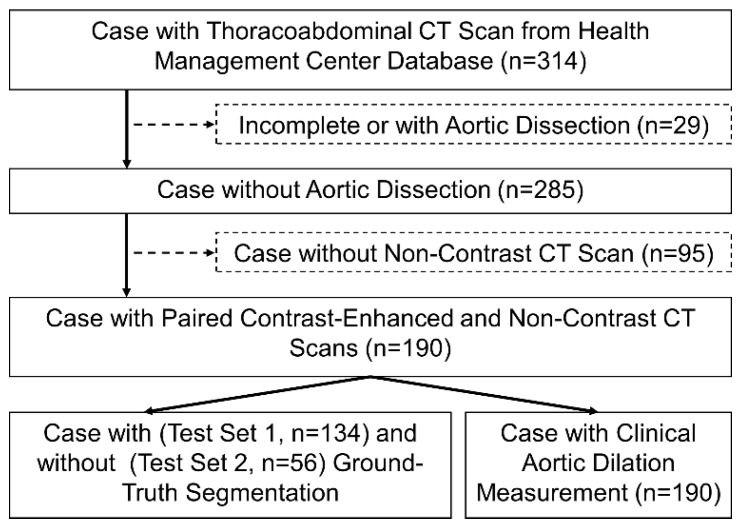
Patient selection flowchart. This figure illustrates the study cohort selection process, including the inclusion and exclusion of cases from the database, the identification of patients with paired contrast-enhanced and non-contrast CT scans, and the final division into test sets with and without ground-truth segmentation.

This study was a retrospective compilation of routine clinical imaging, receiving approval from the Institutional Review Board (IRB) of Cheng Hsin General Hospital (Approval No. [980] 111A-58). The IRB waived informed consent as all data were de-identified before analysis to ensure privacy, with no patient contact or intervention involved. All methods were performed in accordance with relevant guidelines and regulations, including those outlined by the Declaration of Helsinki and the IRB of Cheng Hsin General Hospital. A retrieval of 314 cases from the hospital’s database was conducted between October 2021 and May 2023. Because this was a retrospective validation study, the sample size was determined by the number of eligible cases available in the hospital database during the study period after applying the predefined inclusion and exclusion criteria. Eligible cases were required to have paired contrast-enhanced and non-contrast CT scans acquired in the same session for direct within-patient comparison. Figure 1 depicts the patient selection waterfall diagram.

Within this cohort of 314 cases, 29 exhibited pronounced aortic dissections with discernible true and false lumens. These instances were excluded from our study due to the challenge of distinguishing such cases in CT without contrast. Furthermore, 95 cases were excluded as they only had contrast-enhanced CTs, which did not meet our inclusion criteria. Consequently, 190 cases (380 scans) were incorporated into the study, with an age range between 42–94 (median of 74) and a gender ratio of 98:92 (male to female). Of these, we conducted manual aortic annotations on 134 cases (Test Set 1), while the remaining 56 cases did not receive such annotations (Test Set 2). No cross-validation split was performed, because this study was designed as a retrospective validation of a fixed workflow rather than as a model-training study.

Although all cases were collected at a single center, the dataset reflects routine clinical imaging and includes paired contrast-enhanced and non-contrast CT scans from patients with a broad age range and both sexes, thereby providing a clinically relevant cohort for initial validation. All 190 cases came with clinical records from physicians, who, within their clinical practice, only proceeded with measurements in the presence of an apparent arterial dilation.

### 2.2. CT Imaging Protocol

The CT images were acquired using a dual-source 128-slice multidetector CT scanner (Somatom Definition FLASH, Siemens Healthineers, Erlangen, Germany). Two distinct imaging protocols were implemented. For contrast-enhanced scans, a slice thickness of 1 mm was selected, whilst non-contrast scans were conducted with a 3 mm slice thickness. All scans were performed at a voltage of 120 kVp with automated tube current modulation (CARE Dose4D, Siemens Healthineers). Additionally, pixel spacing for both protocols was the same at 0.65 mm, ensuring consistent spatial resolution across images. During imaging, patients were instructed to fully inhale, hold their breath, and remain still until advised to breathe normally again.

### 2.3. Ground-Truth Segmentation

We used the open-source medical image analysis platform 3D Slicer (version 5.5.0; https://slicer.org/) for manual semi-automated aorta annotation on contrast-enhanced CT images, utilizing the SegmentEditorExtraEffects and SlicerVMTK extensions. The watershed algorithm in the former segmented the aorta based on local histograms, while the Extract Centerline tool automatically detected inlets and outlets to extract the vessel’s centerline. After initial segmentation, inaccurately segmented sections were corrected in 3D space, and the central line of the aorta was calculated. The aorta was then divided into the ascending aorta, aortic arch, and descending aorta based on the brachiocephalic trunk and left subclavian artery positions [36]. This regional subdivision was used for ground-truth annotation and regional validation in the present study, rather than as an automated subdivision output of the workflow. Annotations were performed by medical students and verified by experienced medical professionals.

### 2.4. Automated Aorta Quantification Workflow

**Figure 2:**
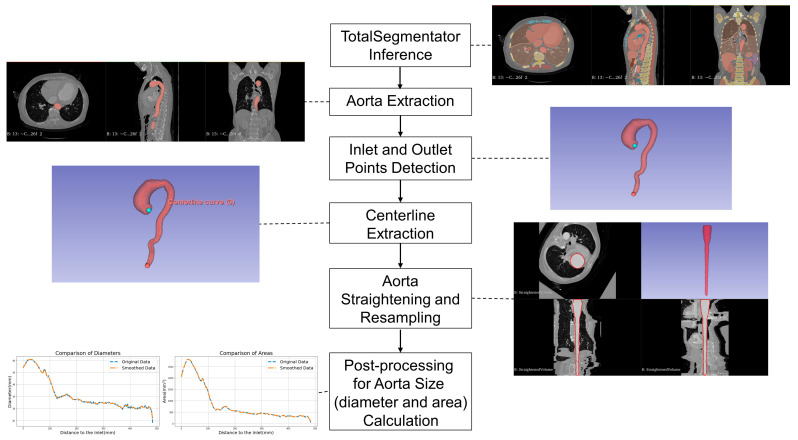
Automated aortic quantification workflow. The workflow comprises six key steps: TotalSegmentator inference, aortic extraction, inlet and outlet detection, vascular centreline extraction, aortic straightening and resampling, and post-processing of aortic dimension calculations. These steps support clinically interpretable aortic quantification by enabling standardized diameter and area measurements that can be compared with region-specific thresholds for aortic dilatation assessment used in this study.

DeepAorta is not a newly designed end-to-end deep neural network architecture; rather, it is an in-house workflow module implemented in 3D Slicer that integrates an existing segmentation model (TotalSegmentator) with geometry-aware post-processing steps for aortic quantification. In this study, contrast-enhanced and non-contrast CT scans from the same patient and imaging session were processed independently using an identical DeepAorta workflow. This paired same-session design enabled a direct within-subject comparison of workflow performance across the two imaging conditions. Our automated aortic quantification process involved several steps (Figure 2). First, TotalSegmentator segmented tissues and organs in the CT images. The aorta was then extracted, and inlet and outlet points were automatically detected using the Extract Centerline module in SlicerVMTK, which identifies candidate endpoints from the vascular topology of the 3D segmentation model. If more than two candidate endpoints were detected, DeepAorta retained the first point and the farthest point from it as the final inlet and outlet for centerline extraction. Next, the vascular centerline was computed, straightened, and resampled to a uniform voxel size of 1 mm^3^. Finally, the aorta’s maximal diameter and cross-sectional area were calculated for each segment using the resampled images. The automated workflow operated on the whole-aorta segmentation, whereas regional comparisons were performed according to the segment definitions described in Section 2.3.

**Figure 3:**
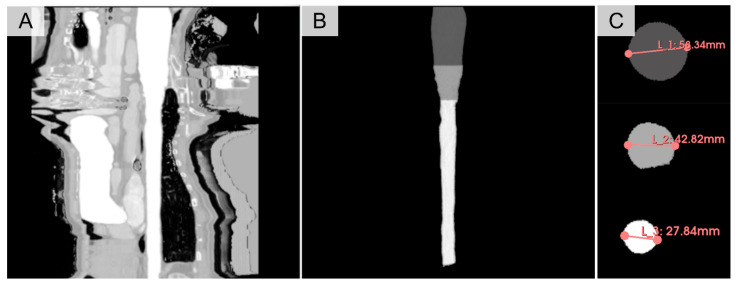
Schematic of aortic quantification. (**A**) Image of the aorta after straightening. (**B**) Label of the straightened aorta. (**C**) Schematic illustration of the area and maximum diameter of different aorta segments.

We define segment levels based on the labelled positions, employing these distinct segments for subsequent AI results assessment. Additionally, leveraging the NumPy package (version 1.22.3), we automate the calculation of the maximum diameter and area size of cross-sections (Figure 3). A smoothing process was applied for the sequence of diameter and area values obtained using the Savitzky–Golay filter from the SciPy package (version 1.10.0), with a window length of 11 and a polynomial order of 2.

We developed an in-house 3D Slicer module, DeepAorta, integrating features from multiple extensions: TotalSegmentator (dcfa71b), SlicerVMTK (144582e), and Sandbox (94e2df0). The Extract Centerline tool in SlicerVMTK enabled automatic detection of the aorta’s inlet and outlet points and centerline calculation, while the Sandbox Curved Planar Reformat extension facilitated vessel straightening and resampling based on the centerline. DeepAorta is an open-source module implemented within 3D Slicer. The computational environment used in this study included the 3D Slicer PyTorch extension, with PyTorch 2.3.0+cu118, TorchVision 0.18.0+cu118, and NVIDIA driver version 560.94. The software was deployed on 3D Slicer version 5.7.0, operating on a Windows 11 system. The primary hardware utilized was a CPU i9-13900K, with 64 GB RAM, and a GPU NVIDIA GeForce RTX 4090 with 24 GB VRAM.

### 2.5. Assessment of Measurements and Statistical Analysis

As previously mentioned, contrast-enhanced CT served as the gold standard for segmentation. We then compared the outcomes of AI-driven automatic quantification on contrast-enhanced and non-contrast CTs. Overlap-based metrics, such as the Dice similarity coefficient and the Intersection over Union (IoU), were used to evaluate segmentation overlap, whereas boundary-distance-based metrics, including the 95% Hausdorff distance and the average symmetric surface distance, were used to assess contour discrepancy [37].

Furthermore, we evaluated the statistical validation of aortic size measurements across the datasets. Pearson correlation coefficients were used to assess the linear association between GT and AI-derived measurements, whereas intraclass correlation coefficients (ICC) were used to evaluate measurement agreement and consistency for aortic diameter and area. In addition, Bland–Altman analysis was performed for the maximum diameter to visualize measurement bias and agreement between GT and AI quantification. Ninety-five percent confidence intervals were reported for the correlation coefficients, ICCs, and ROC analyses.

Lastly, Receiver Operating Characteristic (ROC) analysis was performed to compare routine clinical records and AI quantification in identifying potential aortic dilatation. Because the study did not involve training a new classification model, fold-wise cross-validation metrics were not applicable. Instead, ROC analysis was performed directly on the predefined patient-level test sets, and the optimal sensitivity and specificity are reported in Section 3.4. Specific dilation thresholds were set for different regions [38]: 40 mm for the ascending aorta, 35 mm for the aortic arch, and 30 mm for the descending aorta.

## 3. Results

### 3.1. Aortic Quantification Measurements

Table 1 presents the quantified results of aortic dimensions using GT, AI inference on CT with contrast and without contrast, divided into different segments: the entire aorta, ascending aorta, aortic arch, and descending aorta. The data encompassed maximum, mean, and median diameters/areas for the whole aorta, ascending aorta, aortic arch, and descending aorta.

Under the GT analysis, the whole aorta’s maximum diameter was 38.28 ± 5.42 mm, with the mean and median diameters at 27.17 ± 3.17 mm and 25.31 ± 3.14 mm, respectively. The maximum area recorded was 1047.94 ± 350.20 mm^2^, with mean and median areas at 554.99 ± 136.80 mm^2^ and 461.40 ± 112.76 mm^2^. An increase in aortic dimensions was observed utilizing AI inference on CT with contrast. The whole aorta’s maximum diameter increased to 40.62 ± 5.29 mm, and the maximum area to 1212.65 ± 357.18 mm^2^. Similar increments were noted across the ascending, arch, and descending segments. AI inference on CT without contrast further escalated the dimensions, where the whole aorta’s maximum diameter reached 42.29 ± 5.32 mm, and the maximum area was 1289.10 ± 342.76 mm^2^.

### 3.2. Segmentation Metrics

Table 2 delineates the segmentation metrics between GT and AI inference in CT images (CT_AI_), both with and without contrast.

For GT versus CT_AI_ with contrast, the Dice coefficient ranged from 0.86 ± 0.02 in the descending aorta to 0.91 ± 0.02 in both the ascending aorta and aortic arch. The IoU scores were lowest for the descending aorta at 0.76 ± 0.03 and highest for the ascending aorta at 0.84 ± 0.03. The 95% Hausdorff distance was lowest for the ascending aorta at 2.27 ± 0.53 mm and highest for the descending aorta at 2.63 ± 0.56 mm. The average symmetric surface distance varied minimally, with the lowest observed in the aortic arch at 0.91 ± 0.17 mm and the highest in the descending aorta at 1.44 ± 0.17 mm.

In the comparison between GT and CT_AI_ without contrast, the Dice coefficients indicated lower conformity than CT_AI_ with contrast, ranging from 0.78 ± 0.04 in the descending aorta to 0.87 ± 0.03 in the ascending aorta. The IoU scores mirrored this trend, with the lowest score for the descending aorta at 0.65 ± 0.05 and the highest for the ascending aorta at 0.77 ± 0.05. The 95% Hausdorff distance increased, with the lowest distance in the aortic arch at 3.64 ± 1.05 mm and the highest in the whole aorta at 4.24 ± 0.72 mm. The average symmetric surface distance similarly increased, with the lowest in the aortic arch at 1.45 ± 0.51 mm and the highest in the descending aorta at 2.37 ± 0.35 mm.

### 3.3. Correlation and Consistency Analysis

**Figure 4:**
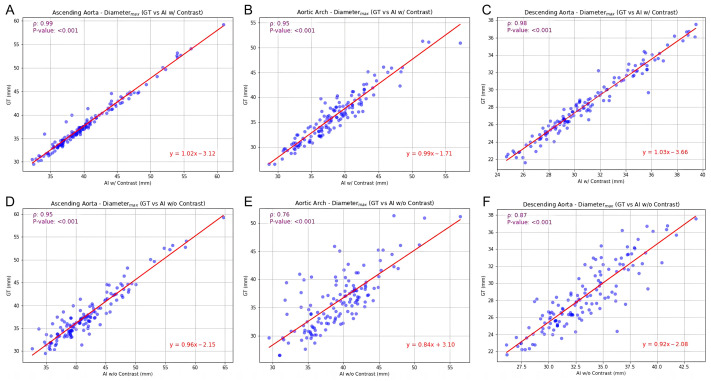
Scatter plot with correlation analysis for maximum diameter. GT versus CT_AI_ with contrast for (**A**) ascending aorta, (**B**) aortic arch, and (**C**) descending aorta. GT versus CT_AI_ without contrast for (**D**) ascending aorta, (**E**) aortic arch and (**F**) descending aorta.

**Figure 5:**
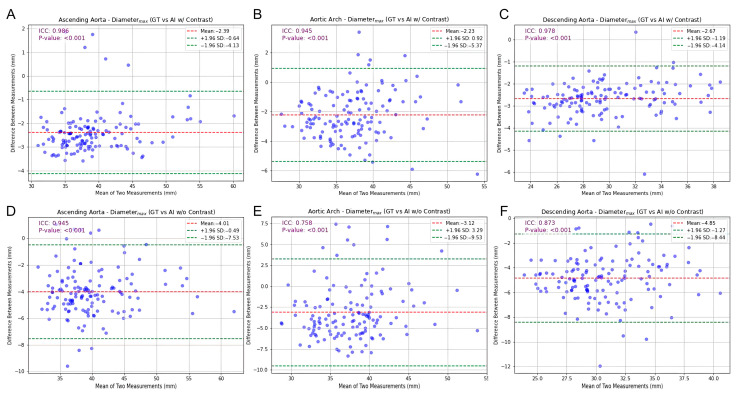
Bland–Altman plot with consistency analysis for maximum diameter. GT versus CT_AI_ with contrast for (**A**) ascending aorta, (**B**) aortic arch, and (**C**) descending aorta. GT versus CT_AI_ without contrast for (**D**) ascending aorta, (**E**) aortic arch, and (**F**) descending aorta.

Figure 4 and Figure 5 depict the correlation coefficients and ICCs for the maximum diameter, illustrating the comparison between GT and CT_AI_ (please refer to Table 3 and Table 4 for comprehensive values).

In CT_AI_ with contrast, there was a high degree of similarity to the GT across all metrics. The correlation coefficient for the maximum diameter of ascending aorta was 0.987 (95% CI: 0.98–0.99). A similar pattern in ICCs, consistently near 1, indicated high reliability in measurements between GT and CT_AI_ with contrast.

The analysis of CT_AI_ without contrast revealed a decline in correlation coefficients and ICCs. The correlation coefficient for the maximum diameter of ascending aorta decreased to 0.945 (95% CI: 0.92–0.96), with a similarly reduced ICC. This trend was accentuated in the aortic arch, where the lowest ICCs were observed. This segment demonstrated the most variation, with correlation coefficients for median diameter dropping as low as 0.678 and ICCs reflecting this reduced agreement (Table 3 and Table 4). The descending aorta, while showing higher overall agreement than the arch in the CT_AI_ without contrast, presented a reduction in correlation coefficients and ICCs compared to the CT_AI_ with contrast.

### 3.4. Receiver Operating Characteristic Analysis

**Figure 6:**
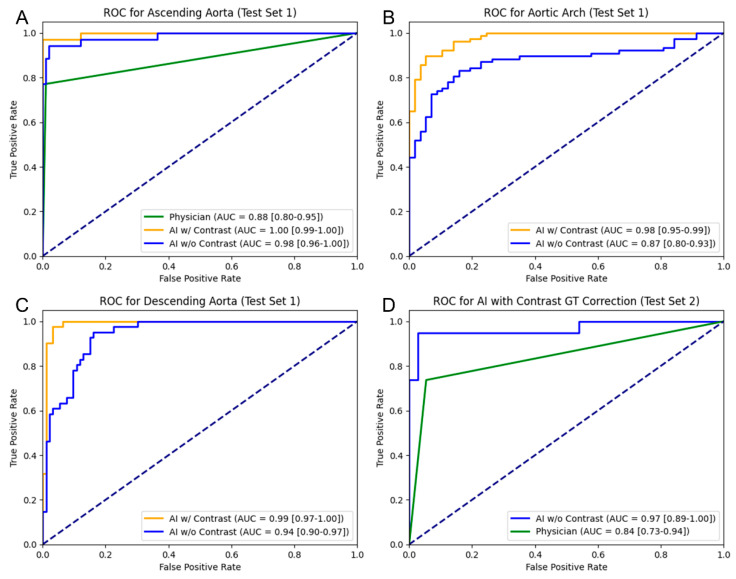
ROC analysis for comparing physicians and CT_AI_ in assessing vascular dilation. (**A**) Ascending aorta, (**B**) aortic arch, and (**C**) descending aorta in Test Set 1. (**D**) Whole aorta in Test Set 2 (using quantification result from CT_AI_ with contrast GT correction as GT).

In the ROC analysis (Figure 6), we compared the efficacy of physicians’ routine clinical records and automated quantification in detecting aortic dilatation. Test Set 1 included 134 cases with GT segmentation, and Test Set 2 contained 56 cases lacking GT. These 190 cases featured routine clinical records from physicians, who recorded dilation measurements in the ascending aorta noted during clinical routines. For Test Set 2, we used the CT_AI_’s quantification results from contrast-enhanced CTs, adjusted via regression correction from Test Set 1, as GT measurements. We were comparing the efficacy of physicians and CT_AI_ in detecting aorta dilation in images without the contrast agent.

For the ascending aorta in Test Set 1, the physician achieved an AUC of 0.88 [0.80–0.95], which was surpassed by the CT_AI_. CT_AI_ with contrast reached the maximum AUC value of 0.997 [0.99–1.00], while that on CT_AI_ without contrast also demonstrated superior accuracy with an AUC of 0.984 [0.96–1.00].

Considering the aortic arch in Test Set 1, there were no clinical records and CT_AI_ with contrast had an AUC of 0.976 [0.95–0.99], whereas the performance of CT_AI_ without contrast declined with an AUC of 0.874 [0.80–0.93]. For the descending aorta in Test Set 1, there were no clinical records, and the CT_AI_ maintained a high level of diagnostic accuracy with contrast (AUC = 0.990 [0.97–1.00]) and without contrast (AUC = 0.942 [0.90–0.97]).

In Test Set 2, which lacked the GT segmentation and involved CT_AI_ with contrast by GT correction as GT measurement for the aorta, CT_AI_ performed robustly without contrast, achieving an AUC of 0.966 [0.89–1.00]. The physician’s AUC in this set was 0.841 [0.73–0.94], lower than the CT_AI_ without contrast. Table 5 summarizes the optimal sensitivity and specificity obtained from the ROC analysis on the predefined patient-level test sets.

## 4. Discussion

We developed a fully automated aortic quantification process using the open-source model and tools, demonstrating high effectiveness in detecting aortic dilation across CT imaging protocols, both with and without a contrast agent. The high degree of correlation and consistency in aortic quantification metrics (close to 1 for the maximum), alongside the impressive AUC scores in detecting vascular dilation (exceeding 0.9 across various segments and imaging protocols), underscore this fact. This reflects the model’s potential to facilitate the early detection and longitudinal monitoring of aortic pathologies. This has the potential to redefine routine clinical imaging processes and, by promoting the early detection of aortic anomalies, to improve the prognoses for affected patients.

Our study observed distinct performance in evaluating aortic segments—ascending aorta, aortic arch, and descending aorta. Our semi-automatic method was based on contrast thresholds that defined the aortic lumen as GT. The AI’s higher measurements stemmed from including the aortic wall, increasing thickness akin to the wall size [39]. The lack of wall contrast in non-contrast CT led to an even larger inclusion area. In clinical practice, diameter assessment on contrast-enhanced studies is often focused on the inner lumen or contrast-opacified vascular boundary. By contrast, in non-contrast CT, measurements that include the outer aortic wall may yield systematically larger absolute values, but may also reflect total aortic size more consistently and therefore may still be useful for serial follow-up when the same imaging modality and measurement approach are used over time. Nonetheless, the correlation and consistency results demonstrated the AI’s exceptional capability in delineating the ascending aorta through contrast-enhanced and non-contrast CTs. In addition, AI performed better in contrast-enhanced CT than non-contrast CT, likely because the ground truth was based on contrast-enhanced CT, and the contrast agent highlighted vascular boundaries, improving AI segmentation and quantification. This challenge is particularly relevant in non-contrast CT, where the boundary between the aortic lumen, wall, and adjacent mediastinal structures is less conspicuous than in contrast-enhanced studies. In addition, the non-contrast protocol used in this study had a thicker slice thickness than the contrast-enhanced protocol, which may further reduce boundary definition and contribute to segmentation variability. Irrespective of using a contrast agent, the AUC scores for all three aortic segments consistently exceeded 0.9 and surpassed clinical records, accentuating the AI’s heightened sensitivity in detecting aortic dilation abnormalities. Nevertheless, despite a slight decline, the AI performance on non-contrast CT remains commendable.

Clinical quantification of the aorta requires specialized software, and manual measurement is time-consuming and subject to certain errors [31]. If the imaging is not specifically conducted to examine the aorta, measurements are only undertaken when a noticeable dilation is observed upon reviewing the images. The AI application for automated aorta quantification saves time and keenly assists and alerts physicians to potential aortic abnormalities in patients. In a potential clinical workflow, such a tool may be integrated as a post-processing or decision-support module to automatically provide aortic measurements and flag possible dilatation for physician review during routine CT interpretation. Our findings suggest that AI-based aortic quantification could be applied in scenarios where contrast agents are contraindicated, thereby broadening the potential role of non-invasive cardiovascular imaging. The ability to delineate aortic structure on non-contrast CT may be particularly valuable for patients with renal impairment or allergies to contrast media, for whom contrast-enhanced studies may be less suitable. From a clinical perspective, this may expand opportunities for opportunistic screening and longitudinal assessment using routinely acquired non-contrast CT examinations. Such an application could support earlier identification of aortic dilatation and facilitate timely clinical attention in daily practice.

Recent research focusing on AI’s application and validation for the automated quantification of the aorta has verified the exceptional accuracy of AI in aortic measurements, demonstrating its feasibility for clinical application [22,23,24,25,26,27,31]. Recent state-of-the-art studies have further shown that automated aortic quantification is feasible across different CT settings, including ECG-gated contrast-enhanced CT angiography, routine chest CT, low-dose chest CT, and non-contrast screening CT [25,26,27,31,33]. For example, Pradella et al. developed a fully automated guideline-compliant workflow for thoracic aortic diameter measurement on ECG-gated CTA [31], whereas Sedghi Gamechi et al. demonstrated automated 3D segmentation and diameter measurement on non-contrast-enhanced CT [33]. Hamelink et al. further validated AI-based thoracic aortic diameter measurement in low-dose chest CT [26], and Graby et al. showed the clinical utility of AI-based thoracic aorta assessment on routine chest CT, including the detection of previously undocumented aortic dilatation [25]. Macruz et al. also demonstrated a fully automatic methodology for thoracic aortic quantification and aneurysm detection on CT [27]. More recent studies in 2025–2026 further support this trend. For example, Heo et al. reported automated aortic diameter measurement in an abdominal CT trauma setting [40], extending AI-based aortic analysis into additional clinical scenarios, whereas the open-source AortaExplorer framework demonstrated high-accuracy end-to-end CTA analysis with validation against manual readings in a very large population [41]. Additionally, AI has proven adept at identifying aortic pathologies like aneurysms and dissections [27,28,29,30], with sensitivity and specificity on par with physicians, further highlighting its potential in clinical usage. Compared with these previous studies, our results similarly support strong clinical feasibility, with high agreement with ground truth in both contrast-enhanced and non-contrast CT, together with AUC values exceeding 0.9 across all aortic segments and imaging protocols. By evaluating the AI performance across contrast-enhanced and non-contrast CTs in identical cases, our research has leveraged paired imaging to affirm AI’s versatility in non-contrast CT settings, contributing to the discipline. Moreover, in contrast to other non-open source or commercial models, our approach is based entirely on the open-source model and tools, enhancing the reproducibility of our proposed methodologies.

Our study is subject to some limitations. Firstly, our validation was conducted on a single machine within a single center. However, the open-source core model we utilized, though not explicitly designed for aortic quantification, has been validated across multiple centers and machines for its accuracy and stability [21]. Therefore, because the present study was based on a single-center dataset acquired on one machine, the broader clinical generalizability of the full DeepAorta workflow remains to be confirmed in future multi-center and multi-scanner studies. Secondly, the clinical records of aortic dilation measurements performed by various physicians were based on subjective assessments, with measurements taken only when aortic dilation was suspected. However, we addressed this issue by delineating the entire aorta and obtaining GT measurements through image processing. Theoretically, this approach should be more accurate and sensitive, a fact that was corroborated by our ROC analysis. Thirdly, our proposed process, centered around the TotalSegmentator as the core model, does not automatically segment the aorta into specific sections or infer results for different aortic landmarks as recommended [4,5]. Instead, our approach relies on statistical methods to identify the position of maximum values and calculate averages and medians from the entire aorta and different segments. Despite these constraints, we have validated the core model’s accuracy in aorta quantification. Future developments based on this model and methodology may train more robust AI systems capable of automatically segmenting distinct aortic sections and detecting landmarks of interest.

Future research should further validate the full DeepAorta workflow in multi-center and multi-scanner datasets to confirm broader clinical generalizability. Additional improvements may include optimization of aortic wall delineation in non-contrast CT, extension to more diverse aortic pathologies, and integration into prospective clinical workflows to evaluate real-world utility in opportunistic screening and longitudinal follow-up.

## 5. Conclusions

In conclusion, this study developed and validated a fully automated aortic quantification workflow based on open-source models and tools, using paired contrast-enhanced and non-contrast CT scans acquired from the same patient during the same imaging session. The proposed workflow demonstrated high agreement with ground truth and strong diagnostic performance across both imaging conditions, supporting its feasibility for automated aortic assessment. These findings suggest that the method may enhance the utility of routine CT examinations and support broader clinical application of aortic quantification in both contrast-enhanced and non-contrast settings. Further multi-center validation is warranted to confirm its generalizability and practical value in routine clinical workflows.

## Figures and Tables

**Table 1:** Aortic dimensions for ground-truth segmentation and AI inference on CT with and without contrast.

Group/Segment	Diameter_max_	Diameter_mean_	Diameter_median_	Area_max_	Area_mean_	Area_median_
Ground Truth (GT)						
Whole Aorta	38.28 ± 5.42	27.17 ± 3.17	25.31 ± 3.14	1047.94 ± 350.20	554.99 ± 136.80	461.40 ± 112.76
Ascending	37.96 ± 5.47	34.45 ± 4.93	35.09 ± 5.18	1045.99 ± 351.13	881.44 ± 281.00	918.89 ± 291.88
Arch	36.07 ± 4.94	30.93 ± 3.60	30.45 ± 3.59	880.52 ± 243.10	666.80 ± 159.30	646.44 ± 158.83
Descending	28.34 ± 3.73	23.70 ± 2.82	23.18 ± 2.85	578.11 ± 150.22	409.09 ± 95.79	391.43 ± 95.50
CT_AI_ with Contrast						
Whole Aorta	40.62 ± 5.29	29.89 ± 3.01	28.07 ± 2.92	1212.65 ± 357.18	687.31 ± 145.57	587.28 ± 122.23
Ascending	40.35 ± 5.31	37.05 ± 4.77	37.65 ± 4.98	1210.50 ± 357.84	1041.52 ± 291.21	1074.78 ± 302.22
Arch	38.30 ± 4.74	32.67 ± 3.34	32.00 ± 3.30	1038.27 ± 253.26	786.01 ± 164.76	751.69 ± 163.93
Descending	31.00 ± 3.54	26.64 ± 2.72	26.19 ± 2.71	709.88 ± 163.62	533.40 ± 108.88	513.49 ± 107.23
CT_AI_ without Contrast						
Whole Aorta	42.29 ± 5.32	31.96 ± 2.97	30.40 ± 2.99	1289.10 ± 342.76	768.80 ± 144.65	675.77 ± 131.73
Ascending	41.98 ± 5.41	38.42 ± 4.76	39.23 ± 4.73	1286.14 ± 343.61	1098.55 ± 273.96	1142.13 ± 275.68
Arch	39.20 ± 4.48	34.29 ± 3.72	33.90 ± 4.04	1081.55 ± 243.92	859.04 ± 188.75	840.79 ± 202.72
Descending	33.19 ± 3.55	29.06 ± 2.78	28.79 ± 2.81	798.20 ± 172.00	625.38 ± 119.07	611.88 ± 119.77

Note: GT denotes ground truth; CT_AI_ denotes AI inference in computed tomography images. All diameter measurements are expressed in millimeters (mm), and area measurements are in square millimeters (mm^2^). GT measurements were lumen-based, whereas AI measurements may include the aortic wall, which may contribute to the size difference.

**Table 2:** Segmentation metrics for ground-truth segmentation and AI inference on CT with and without contrast.

Segment	Dice	IoU	95% Hausdorff Distance (mm)	Average Symmetric Surface Distance (mm)
GT vs. CT_AI_ with Contrast				
Whole Aorta	0.89 ± 0.02	0.80 ± 0.03	2.53 ± 0.46	1.40 ± 0.15
Ascending	0.91 ± 0.02	0.84 ± 0.03	2.27 ± 0.53	1.15 ± 0.16
Arch	0.91 ± 0.02	0.83 ± 0.04	2.30 ± 0.55	0.91 ± 0.17
Descending	0.86 ± 0.02	0.76 ± 0.03	2.63 ± 0.56	1.44 ± 0.17
GT vs. CT_AI_ without Contrast				
Whole Aorta	0.82 ± 0.03	0.70 ± 0.05	4.24 ± 0.72	2.25 ± 0.32
Ascending	0.87 ± 0.03	0.77 ± 0.05	3.95 ± 1.14	1.76 ± 0.40
Arch	0.85 ± 0.05	0.74 ± 0.07	3.64 ± 1.05	1.45 ± 0.51
Descending	0.78 ± 0.04	0.65 ± 0.05	4.15 ± 0.68	2.37 ± 0.35

Note: GT denotes ground truth; CT_AI_ denotes AI inference in computed tomography images.

**Table 3:** Correlation coefficients for aortic dimensions between ground truth and AI inference.

Segment	Diameter_max_	Diameter_mean_	Diameter_median_	Area_max_	Area_mean_	Area_median_
GT vs. CT_AI_ with Contrast						
Ascending	0.987 * [0.98–0.99]	0.998 * [0.99–1.00]	0.998 * [0.99–1.00]	0.997 * [0.99–1.00]	0.997 * [0.99–1.00]	0.997 * [0.99–1.00]
Arch	0.946 * [0.92–0.96]	0.976 * [0.97–0.98]	0.962 * [0.95–0.97]	0.989 * [0.98–0.99]	0.982 * [0.97–0.99]	0.979 * [0.97–0.99]
Descending	0.980 * [0.97–0.99]	0.993 * [0.99–0.99]	0.989 * [0.99–0.99]	0.983 * [0.98–0.99]	0.986 * [0.98–0.99]	0.987 * [0.98–0.99]
GT vs. CT_AI_ without Contrast						
Ascending	0.945 * [0.92–0.96]	0.959 * [0.94–0.97]	0.972 * [0.96–0.98]	0.980 * [0.97–0.99]	0.962 * [0.95–0.97]	0.968 * [0.96–0.98]
Arch	0.762 * [0.68–0.82]	0.746 * [0.66–0.81]	0.678 * [0.57–0.76]	0.813 * [0.75–0.86]	0.722 * [0.63–0.79]	0.642 * [0.53–0.73]
Descending	0.874 * [0.83–0.91]	0.947 * [0.93–0.96]	0.929 * [0.90–0.95]	0.890 * [0.85–0.92]	0.950 * [0.93–0.96]	0.942 * [0.92–0.96]

Note: GT, ground truth; CT_AI_, AI inference in computed tomography images. * *p* < 0.001.

**Table 4:** Intraclass correlation coefficients for aortic dimensions between ground truth and AI inference.

Segment	Diameter_max_	Diameter_mean_	Diameter_median_	Area_max_	Area_mean_	Area_median_
GT vs. CT_AI_ with Contrast						
Ascending	0.986 * [0.98–0.99]	0.997 * [0.99–1.00]	0.997 * [0.99–1.00]	0.997 * [0.99–1.00]	0.996 * [0.99–1.00]	0.996 * [0.99–1.00]
Arch	0.945 * [0.92–0.96]	0.973 * [0.96–0.98]	0.958 * [0.94–0.97]	0.988 * [0.98–0.99]	0.982 * [0.97–0.99]	0.979 * [0.97–0.98]
Descending	0.978 * [0.97–0.98]	0.992 * [0.99–0.99]	0.988 * [0.98–0.99]	0.980 * [0.97–0.99]	0.978 * [0.97–0.98]	0.981 * [0.97–0.99]
GT vs. CT_AI_ without Contrast						
Ascending	0.945 * [0.92–0.96]	0.958 * [0.94–0.97]	0.968 * [0.96–0.98]	0.979 * [0.97–0.99]	0.962 * [0.95–0.97]	0.967 * [0.95–0.98]
Arch	0.758 * [0.68–0.82]	0.746 * [0.66–0.81]	0.673 * [0.57–0.76]	0.813 * [0.75–0.86]	0.711 * [0.62–0.79]	0.623 * [0.51–0.72]
Descending	0.873 * [0.83–0.91]	0.947 * [0.93–0.96]	0.929 * [0.90–0.95]	0.882 * [0.84–0.91]	0.927 * [0.90–0.95]	0.918 * [0.89–0.94]

Note: GT, ground truth; CT_AI_, AI inference in computed tomography images. * *p* < 0.001.

**Table 5:** Optimal metrics for receiver operating characteristic analysis.

Segment	Optimal Sensitivity	Optimal Specificity	AUC
Ascending Aorta			
Routine Records by Physicians	77%	99%	0.880 [0.80–0.95]
CT_AI_ with Contrast	97%	100%	0.997 [0.99–1.00]
CT_AI_ without Contrast	94%	98%	0.984 [0.96–1.00]
Aortic Arch			
CT_AI_ with Contrast	90%	95%	0.976 [0.95–0.99]
CT_AI_ without Contrast	83%	84%	0.874 [0.80–0.93]
Descending Aorta			
CT_AI_ with Contrast	98%	97%	0.990 [0.97–1.00]
CT_AI_ without Contrast	95%	84%	0.942 [0.90–0.97]
Whole AortaCTAI with Contrast after GT Correction			
Routine Records by Physicians	74%	85%	0.841 [0.73–0.94]
CT_AI_ without Contrast	95%	97%	0.966 [0.89–1.00]

Note: GT, ground truth; CT_AI_, AI inference in computed tomography images.

## Data Availability

The source code supporting the findings of this study is openly available at: https://github.com/EternityJH/DeepAorta (accessed on 14 April 2026). The datasets generated and/or analyzed during the current study are not publicly available due to restrictions imposed by the approved protocol from the IRB of Cheng Hsin General Hospital (Approval No. [980] 111A-58), which did not include provisions for data sharing. All data were retrospectively collected and fully de-identified to protect patient privacy. Requests for access to the data can be made by contacting the corresponding author, subject to institutional approvals.
